# Impairment of Quality of Life Associated With Lifetime Diagnosis of Post-traumatic Stress Disorder in Women - A National Survey in Italy

**DOI:** 10.2174/1745017901915010038

**Published:** 2019-02-28

**Authors:** Federica Sancassiani, Claudia Carmassi, Ferdinando Romano, Matteo Balestrieri, Filippo Caraci, Guido Di Sciascio, Filippo Drago, Carlo Faravelli, Maria Carolina Hardoy, Maria Francesca Moro, Rita Roncone, Antonio Preti, Liliana Dell’Osso

**Affiliations:** 1 Department of Medical Sciences and Public Health, University of Cagliari, Cagliari, Italy;; 2 University of Pisa ,Pisa, Italy;; 3 University of Roma “La Sapienza”, Rome, Italy;; 4 University of Udine,Udine, Italy;; 5 University of Catania, Catania, Italy;; 6 University of Bari, Bari, Italy;; 7 University of Florence, Florence, Italy;; 8 Mailman School of Public Health Columbia University, New York, NY 10027, USA; 9 University of L’Aquila, L’Aquila, Italy

**Keywords:** Quality of life, PTSD, Psychiatric comorlridity, Women health, Community survey, Mood disorder

## Abstract

**Introduction:**

The aim of the study was to measure the lifetime prevalence of Post-Traumatic Stress Disorder (PTSD) among women of an Italian community sample, the comorbidity of PTSD with mood and anxiety disorders and the burden attributable to PTSD in worsening the Quality of Life (QoL).

**Methods:**

Community survey on a sample of 1961 adult women randomly selected. Tools: psychiatric clinical interview ANTAS partially derived from the SCID-DSM-IV, administered by psychologists or medical doctors; Short Form Health Survey (SF-12); Mood Disorder Questionnaire (MDQ).

**Results:**

Lifetime prevalence of PTSD in women was 1.3%, (1.4% in<45 years aged, 1.3% in >44 years aged; p=0.8). In order of risk of comorbidity, PTSD was associated with: Bipolar Spectrum Disorders (MDQ+), Panic Disorders (PD) and Major Depressive Disorder (MDD). People with PTSD showed an SF-12 mean score lower than women of the same sample without PTSD (standardized by gender and age), with a mean difference (attributable burden) of 3.9±0.9 similarly to MDD and Eating Disorders and higher than PD. Among the analyzed nonpsychiatric diseases, Multiple Sclerosis and Carotid Atherosclerosis showed a higher burden in impairing QoL than PTSD; Wilson’s Disease showed a similar burden and Celiac Disease was found less impairing on QoL than PTSD.

**Conclusion:**

The attributable burden in worsening women’ perceived QoL due to a lifetime diagnosis of PTSD was found comparable to those caused by MDD, Eating Disorders or by neurological condition such as Wilson’s Disease. The comorbidity of PTSD with Bipolar Spectrum Disorders was remarkable, even further studies are needed to clarify the direction of causality.

## 1 INTRODUCTION

Epidemiological studies on the frequency of mental disorders do not always report prevalence rates of PTSD, espe cially when they did not involve populations damaged by natural catastrophes or military conflicts [1-4]. However, surveys as NCS, WMH and ESEMED (the European section of WMH) have reported it [5-7]. In WMH study, the lifetime prevalence of PTSD was found quite similar in South Africa (2.3%) [8], Spain (2.2%) [9] and Italy (2.4%) [10], while it was lower in Japan (1.3%) [11] and sensibly higher in Northern Ireland (8.8%) [12].

The WMH survey highlights that in a given population that has been affected by natural disasters, conflicts or wars, these events seemed to influence the distribution of PTSD along with other factors. Otherwise, these populations are usually involved to carry out studies on PTSD to early detect and treat PTSD and/or other trauma-related diseases.

However, it is known that also other kind of traumatic environmental factors, such as childhood trauma, are associated with a higher risk of several psychiatric disorders as PTSD because of their role in making the brain more vulnerable to subsequent trauma [13]. Furthermore, the amount of previous traumatic events impacts on the severity and complexity of PTSD [13, 14] and the rates of the disorders were found higher among women in almost all the sites where the surveys were conducted [1-14]. In particular, women outnumber men by an average of 2:1 [15-17] and seem to be a more vulnerable population [14-17].

For these reasons, it would be very interesting to understand how the PTSD can impair social life and the Quality of Life (QoL) of the subjects suffering from PTSD, also in communities not affected by natural disasters or wars [18]. When related to the general population, this figure would create a picture of the social impact of the disorder, while at the individual level, it would allow to understand the degree of subjective suffering, particularly among women.

The objectives of our study were:

1. To estimate the lifetime prevalence of PTSD among women of a community sample from six Italian regions;
2. To measure the comorbidity of PTSD with other psychiatric disorders;
3. To measure the QoL of women with a lifetime PTSD diagnosis and to compare it with the QoL of women without PTSD;
4. To estimate the burden attributable to the PTSD in worsening QoL and to compare it with the burden due to other disorders and diseases.

It is interesting to study QoL impairment in PTSD because of the subjective perception of QoL involved in both the physical and the psychological components of wellbeing, including satisfaction about sleep and rest, energy, vitality, sociality and perceived pain [19]. Furthermore, QoL has become a measure of relevance about the outcome in chronic diseases, especially in those that require long-term treatments that greatly impact on daily life [20-22].

## 2 METHODS

### 2.1 Design

Community survey carried out with face-to-face semi-structured interviews conducted by trained clinicians (medical doctors or clinical psychologists with at least two years of experience in psychiatry).

### 2.2 Recruitment and Study Sample

The sample (females ≥18 years old) was randomly selected from municipal records of seven different Italian regions, including urban and rural areas in each region. The methodology of sampling, recruitment, management and monitoring of the study has been detailed previously [23, 24].

### 2.3 Tools and Assessment

The following tools have been administered:

1. Ad-hoc form, administered and validated in previous surveys to collect demographic data [23, 24];
2. The“Advanced Neuropsychiatric Tools and Assessment Schedule” (ANTAS) [23, 24], a semi-structured psychiatric clinical interview-derived from the Structured Clinical Interview for DSM-IV, Non-patient version(SCID-NP) for psychiatric diagnoses according to DSM-IV [25]. High reliability (Cohen’s K = 0.85) between ANTAS and SCID-NP was found [23, 24].
3. The Italian version [26] of the Short Form Health Survey (SF-12) [27] was used to measure the QoL. The SF-12 includes the following dimensions: physical activity, physical health, mental health, pain, general health, vitality and social activity. SF-12 measures the perceived QoL in the month before the interview, with a higher score showing better QoL.
4. Bipolar Spectrum Symptoms were detected by means of the Italian version of the Mood Disorder Questionnaire (MDQ) [28], using 7/8 as a cut-off score.

### 2.4 Ethical Aspects

The ethical committee of the Italian National Health Institute (“IstitutoSuperioredellaSanità”) in Rome approved the study protocol. Each candidate signed the informed consent form. The study was carried out according to the Helsinki Declaration.

### 2.5 Data Analysis

According to DSM-IV criteria [29], lifetime prevalence for PTSD was calculated in the women sample, as well as the co-morbidity with PTSD and main Psychiatric DSM-IV Disorders. The odds ratios of association were calculated for each psychiatric disorder (univariate analysis). Statistical significance was calculated using the χ^2^ test in 2 x 2 tables.

The “attributable burden” due to PTSD was calculated as the difference between the QoL of those with a diagnosis of PTSD (score at SF-12) and the level of QoL (score at SF-12) in a sample of women of the same database without PTSD, drawn after a block randomization by age and sex according to the sample of women with PTSD.

The attributable burden in worsening QoL due to PTSD compared to the attributable burden calculated for other diseases in previous studies, using the same database to draw the controls from (Major Depressive Disorders [23], Eating Disorder [30], Panic Disorder [31], Wilson’s Disease [32], Multiple Sclerosis [33], Carotid Atherosclerosis [34], and Celiac Disease [35].

## 3 RESULTS

The interviewed women were 1961 [23]. As shown in Table **1**, the PTSD lifetime prevalence was 1.3%, without any difference according to age: 1.4% were among the <45 years aged, 1.3% were among the >44 years aged (χ^2^=0.06, p=0.808, OR <45 =1.1 (CI 95% 0.5-2.5).

Table **2** shows the rates of comorbid mood and anxiety disorders in women with PTSD. In order of odds ratio, PTSD was associated with: Bipolar Spectrum Disorders (identified as MDQ positives) OR=14.98 (CI95% 5.0-42.1), Panic Disorders (PD)OR=12.33 (CI 95% 4.9-9.4) and Major Depressive Disorder (MDD) OR=2.89. (CI95% 1.1-7.4).

As shown in Table **3**, people with PTSD showed an SF-12 mean score lower than women in the same sample without PTSD (standardized by sex and age), with a mean ±sd difference (attributable burden) of 3.9±0.9. This burden was similar to that due to Major Depressive Disorders, Eating Disorders and Wilson Disease, higher than the burden due to Celiac Disease and Panic Disorder, but lower than the burden due to Multiple Sclerosis and Carotid Atherosclerosis.

## 4 DISCUSSION

The study pointed out a lower PTSD frequency than similar studies conducted on populations damaged by disrupting natural disasters or traumatizing conflicts. However, the PTSD lifetime prevalence found in this study is lower than the rates found in the WMH study in all the sites (including Italy) [8-10], with the exception of Japan [11]. However, it should be emphasized that WMH survey used rigid diagnostic interviews, with only YES/NO answers, conducted by lay interviewers [36, 37], while our study has adopted semi-structured interviews carried out by clinicians [23]. This second method has shown that disorders such as Major Depressive Disorder [23] and Obsessive Compulsive Disorder [38] have lower frequencies with respect to the WMH. It is therefore likely that a similar trend occurs in relation to PTSD. However, the diagnostic methodology is closer to the one adopted in clinical practice.

Notably, this study points out the rate of co-morbid mood and anxiety disorders in women with PTSD. In order of odds ratio, PTSD was firstly associated with Bipolar Spectrum Disorders. This association confirms previous observations [39, 40] and questions the notion that PTSD is the result of contingent etiological factors only. However, the cross-sectional design of this survey does not allow to clarify the direction of causality: whether it is exposure to stress that can trigger a possible vulnerability for Bipolar Spectrum Disorders, or if the presence of a Bipolar Spectrum Disorder, through hyper-exploratory behavior, exposes to a higher risk to PTSD, or if there is an interaction between the two factors.

In the present study, women with PTSD show a serious impairment of their QoL. PTSD is responsible for lowering the perception of QoL to a degree similar to other serious psychiatric disorders as Major Depressive Disorder [23] and Eating Disorders [30] but to a greater degree than Panic Disorder [31], however disabling it may be.

The comparison with non-psychiatric disorders gives an idea of the impact of PTSD on people's lives, considering that PTSD results in a compromised QoL that is greater than Celiac Disease [35], similar to a highly disabling disease such as Wilson's disease [32] but, however, lower than the one determined by Multiple Sclerosis [33] and Carotid Atherosclerosis [34].

The above-cited case-control studies focused on psychiatric disorders (Major Depressive Disorder, Panic Disorder, Eating Disorders) have adopted the same methodology as this study. In fact the “cases” were drawn from the same database of this study (the “cases” in each study were all prevalent cases of the diagnosis under examination) and the “healthy” controls (people in the community without the analyzed disorder) were drawn from the same community sample after randomization matching by blocks according to age and sex with respect to the “cases”.Similarly, in the case-control studies focused on non-psychiatric diagnoses (Carotid Atherosclerosis, Wilson's Disease, Multiple Sclerosis, Celiac Disease), the “healthy” controls (people in the community without the analyzed disease) were drawn from the same database of the present study. Therefore, the score on SF-12 measuring the QoL of controls (the comparative criterion) was exactly the same in the various surveys (but with the control sample’s being standardized by age and sex with each specific case sample). Consequently, the attributable burden for each specific disorder or disease is well comparable to the others. This methodology allows us to reliably define the degree of the impact of PTSD, a disorder that implies an attributable burden similar to a well-known disabling nonpsychiatric disease such as Wilson’s disease. It is important to underline that a potentially chronic disorder such as PTSD which is not always chronic, however worsens the QoL equal to disorders such as Wilson's disease which, due to their characteristics, are often chronic.

Finally, it is interesting to note that SF-12 measures the QoL in the last month. When considering the sample of all the subjects who have a lifetime diagnosis of PTSD, the mean score of the SF-12 in this sample is determined by the score of those who have been diagnosed 5 years earlier and are now asymptomatic, those who have been diagnosed 8 years earlier and are now still suffering, as well as those who had the first episode a month before the interview. The chronic cases will show a worsening of the QoLat each point in their life.

From this point of view, the study confirms that PTSD is a disorder that strongly impacts the QoL of people affected.

## 5 LIMITATIONS

This research has focused on the secondary aim of a community survey that had various main objectives. The degree of disability generated by psychiatric disorders should be measured by taking into account their comorbidity with other medical diseases, and the response to treatments as co-determinants. These variables would be investigated by case-control studies. Therefore, the results of this survey are to be considered preliminary and of heuristic value.

## CONCLUSION

The attributable burden on the impairment of QoL due to a lifetime diagnosis of PTSD was found relevant with respect to the average of QoL of people in the community. The degree of this burden is the same if compared with other psychiatric diagnoses such as Major Depressive Disorder and Eating Disorders, or with a neurological condition as Wilson’s Disease. The PTSD does not reach the degree of QoL worsening as Multiple Sclerosis. The significant association with the Bipolar Spectrum Disorders questions the notion that PTSD is the result of contingent etiological factors only, even further studies are needed to clarify the direction of causality.

## Figures and Tables

**Table 1:** PTSD Lifetime Prevalence Rates.

-	**Female Interviewer**	**<45 Years Age**	**>44 Years Age**
Total	1961	859 (43.8%)	1102 (56.2%)
With PTSD diagnosis	26 (1.3%)	12 (1.4%)*	14 (1.3%)*

*χ^2^ =0.06, p=0.808, OR <45 =1.1 (CI 95% 0.5-2.5)

**Table 2:** Comorbidity between PTSD and other Psychiatric Disorders.

-	**% of PTSD** **with Comorbidity**	**χ^2^**	**p**	**OR**	**CI 95%**
Major Depressive Disorder	26.9	6.13	0.013	2.89	1.1-7.4
Bipolar Spectrum Disorders (MDQ+)	23.1	52.14	<0.0001	14.98	5.0-42.1
Panic Disorder	34.6	51.17	<0.0001	12.33	4.9-9.4
Social Phobia	3.8	1.82*	0.178	12.86	0.6-115.1

*with Yates correction

**Table 3:** Attributable Burden Due to PTSD in Decreasing QoL and Comparison with other Disorders.

**Disorders** **[Studyreference]**	**SF-12** **(Mean±sd)**	Attributable Impairment of QoL Due to the Disorder*	**Comparison with** **PTSD** **(ANOVA 1 Way)**
Major Depressive Disorder [23]	33.8±9.2	5.6±3.6 (N=37)	df 1,58,59 F=2.234; p=0.140
Multiple Sclerosis [33]	32.1±7.6	7.0±3.5 (N=201)	df 1,225,226 F=18.544;p<0.0001
Wilson Disease [32]	33.8±9.0	4.4±1.7 (N=23)	df 1,47,48 F=0.719; p=0.401
Eating Disorders [30]	34.9±6.2	4.4±6.6 (N=60)	df 1,81,82 F=0.001; p=0.999
Panic Disorder [31]	35.5±4.6	2.9±0.9 (N=123)	df 1,144,145 F=38.6; p<0.0001
Celiac Disease [35]	35.83±5.72	2.4±1.0 (N=60)	df 1,84,85 F=40.8; p<0.0001
Carotid Atherosclerosis [34]	30.6±8.1	6.2±5.0 (N=46)	df 1,70,71 F=5.34; p=0.024
PTSD	36.3±6.1	3.9±0.9 (N=26)	-

*Difference on SF-12 score between a standardized sample without disorder and a sample with a given disorder

## References

[1] Regier D.A., Narrow W.E., Rae D.S. (1990). The epidemiology of anxiety disorders: the Epidemiologic Catchment Area (ECA) experience.. J. Psychiatr. Res..

[2] Breslau N., Kessler R.C., Chilcoat H.D., Schultz L.R., Davis G.C., Andreski P. (1998). Trauma and posttraumatic stress disorder in the community: the 1996 Detroit Area Survey of Trauma.. Arch. Gen. Psychiatry.

[3] Carta M.G., Oumar F.W., Moro M.F., Moro D., Preti A., Mereu A., Bhugra D. (2013). Trauma- and stressor related disorders in the tuareg refugees of a cAMP in burkina faso.. Clin. Pract. Epidemiol. Ment. Health.

[4] Carta M.G., Moro D., Wallet Oumar F., Moro M.F., Pintus M., Pintus E., Minerba L., Sancassiani F., Pascolo-Fabrici E., Preti A., Bhugra D.K. (2018). A Follow-Up on Psychiatric Symptoms and Post-Traumatic Stress Disorders in Tuareg Refugees in Burkina Faso.. Front. Psychiatry.

[5] Alonso J., Angermeyer M.C., Bernert S., Bruffaerts R., Brugha T.S., Bryson H., de Girolamo G., Graaf R., Demyttenaere K., Gasquet I., Haro J.M., Katz S.J., Kessler R.C., Kovess V., Lépine J.P., Ormel J., Polidori G., Russo L.J., Vilagut G., Almansa J., Arbabzadeh-Bouchez S., Autonell J., Bernal M., Buist-Bouwman M.A., Codony M., Domingo-Salvany A., Ferrer M., Joo S.S., Martínez-Alonso M., Matschinger H., Mazzi F., Morgan Z., Morosini P., Palacín C., Romera B., Taub N., Vollebergh W.A., ESEMeD/MHEDEA 2000Investigators (2004). EuropeanStudy of the Epidemiology of Mental Disorders (ESEMeD) Project. Prevalence of mental disorders in Europe: results from the EuropeanStudy of the Epidemiology of Mental Disorders (ESEMeD) project.. Acta Psychiatr. Scand. Suppl..

[6] De Girolamo G., Polidori G., Morosini P. (2005). La prevalenza dei disturbi mentali in Italia. Il Progetto ESEMED-WMH. Una sintesi. ISS, Roma. http://www.epicentro.iss.it/temi/mentale/esemed.pdf.

[7] Kessler R.C., Berglund P., Demler O., Jin R., Merikangas K.R., Walters E.E. (2005). Lifetime prevalence and age-of-onset distributions of DSM-IV disorders in the National Comorbidity Survey Replication.. Arch. Gen. Psychiatry.

[8] Atwoli L., Stein D.J., Williams D.R., Mclaughlin K.A., Petukhova M., Kessler R.C., Koenen K.C. (2013). Trauma and posttraumatic stress disorder in South Africa: analysis from the South African Stress and Health Study.. BMC Psychiatry.

[9] Olaya B., Alonso J., Atwoli L., Kessler R.C., Vilagut G., Haro J.M. (2015). Association between traumatic events and post-traumatic stress disorder: results from the ESEMeD-Spain study.. Epidemiol. Psychiatr. Sci..

[10] Carmassi C., Dell’Osso L., Manni C., Candini V., Dagani J., Iozzino L., Koenen K.C., de Girolamo G. (2014). Frequency of trauma exposure and Post-Traumatic Stress Disorder in Italy: analysis from the World Mental Health Survey Initiative.. J. Psychiatr. Res..

[11] Kawakami N., Tsuchiya M., Umeda M., Koenen K.C., Kessler R.C. (2014). WorldMental HealthSurveyJapan. Trauma and posttraumatic stress disorder in Japan: resultsfrom the WorldMental HealthJapanSurvey.. J. Psychiatr. Res..

[12] Ferry F., Bunting B., Murphy S., O’Neill S., Stein D., Koenen K. (2014). Traumatic events and their relative PTSD burden in Northern Ireland: a consideration of the impact of the ‘Troubles’.. Soc. Psychiatry Psychiatr. Epidemiol..

[13] Kim J.S., Lee S.H. (2016). Influence of interactions between genes and childhood trauma on refractoriness in psychiatric disorders.. Prog. Neuropsychopharmacol. Biol. Psychiatry.

[14] Karam E.G., Friedman M.J., Hill E.D., Kessler R.C., McLaughlin K.A., Petukhova M., Sampson L., Shahly V., Angermeyer M.C., Bromet E.J., de Girolamo G., de Graaf R., Demyttenaere K., Ferry F., Florescu S.E., Haro J.M., He Y., Karam A.N., Kawakami N., Kovess-Masfety V., Medina-Mora M.E., Browne M.A., Posada-Villa J.A., Shalev A.Y., Stein D.J., Viana M.C., Zarkov Z., Koenen K.C. (2014). Cumulative traumas and risk thresholds: 12-month PTSD in the World Mental Health (WMH) surveys.. Depress. Anxiety.

[15] Kimerling R., Allen M.C., Duncan L.E. (2018). Chromosomes to Social Contexts: Sex and Gender Differences in PTSD.. Curr. Psychiatry Rep..

[16] Goldstein R.B., Smith S.M., Chou S.P., Saha T.D., Jung J., Zhang H., Pickering R.P., Ruan W.J., Huang B., Grant B.F. (2016). The epidemiology of DSM-5 posttraumatic stress disorder in the United States: results from the National Epidemiologic Survey on Alcohol and Related Conditions-III.. Soc. Psychiatry Psychiatr. Epidemiol..

[17] Blanco C, Hoertel N, Wall MM, Franco S, Peyre H, Neria Y, Helpman L, Limosin F (2018). Toward Understanding Sex Differences inthe Prevalence of Posttraumatic Stress Disorder: Results rom the National Epidemiologic Survey on Alcohol and Related Conditions.. J Clin Psychiatry..

[18] Wittchen H.U., Heinig I., Beesdo-Baum K. (2014). [Anxiety disorders in DSM-5: an overview on changes in structure and content].. Nervenarzt.

[19] The WHOQOL Group (1995). The World Health Organization Quality of Life assessment (WHOQOL): position paper from the World Health Organization.. Soc. Sci. Med..

[20] Mantovani G., Astara G., Lampis B., Bianchi A., Curreli L., Orrù W., Carta M.G., Carpiniello B., Contu P., Rudas N. (1996). Evaluation by multidimensional instruments of health-related quality of life of elderly cancer patients undergoing three different “psychosocial” treatment approaches. A randomized clinical trial.. Support. Care Cancer.

[21] Mathers C.D., Stein C., Ma Fat D., Rao C., Inoue M., Tomijima N., Bernard C., Lopez A.D., Murray C.J.L. (2002). Global Burden of Disease 2000: Version 2 methods and results. Global Programme on Evidence for Health Policy Discussion Paper No. 50..

[22] Roberts J., Lenton P., Keetharuth A.D., Brazier J. (2014). Quality of life impact of mental health conditions in England: results from the adult psychiatric morbidity surveys.. Health Qual. Life Outcomes.

[23] Carta M.G., Aguglia E., Bocchetta A., Balestrieri M., Caraci F., Casacchia M., Dell’osso L., Sciascio G.D., Drago F., Faravelli C., Lecca M.E., Moro M.F., Morosini P.L., Nardini M., Palumbo G., Hardoy M.C. (2010). The use of antidepressant drugs and the lifetime prevalence of major depressive disorders in Italy.. Clin. Pract. Epidemiol. Ment. Health.

[24] Carta M.G., Aguglia E., Balestrieri M., Calabrese J.R., Caraci F., Dell’Osso L., Di Sciascio G., Drago F., Faravelli C., Lecca M.E., Moro M.F., Nardini M., Palumbo G., Hardoy M.C. (2012). The lifetime prevalence of bipolar disorders and the use of antidepressant drugs in bipolar depression in Italy.. J. Affect. Disord..

[25] First M., Spitzer R., Gibbon M., Williams J. (1997). Structured clinical interview for DSM-IV axis I disorders, research version, non-patient edition (SCID-I/NP)..

[26] Apolone G., Mosconi P., Quattrociocchi L., Gianicolo E.A., Groth N., Ware J.E. (2001). Questionario sullo stato di salute SF-12 Versione Italiana..

[27] Ware J., Kosinski M., Keller S.D.A. (1996). A 12-Item Short-Form Health Survey: construction of scales and preliminary tests of reliability and validity.. Med. Care.

[28] Hardoy M.C., Cadeddu M., Murru A., Dell’Osso B., Carpiniello B., Morosini P.L., Calabrese J.R., Carta M.G. (2005). Validation of the Italian version of the “Mood Disorder Questionnaire” for the screening of bipolar disorders.. Clin. Pract. Epidemiol. Ment. Health.

[29] American Psychiatric Association (APA) (1994). Diagnostic And Statistical Manual of Mental Disorders: DSM-IV..

[30] Carta M.G., Preti A., Moro M.F., Aguglia E., Balestrieri M., Caraci F., Dell’Osso L., Di Sciascio G., Drago F., Faravelli C., Hardoy M.C., D’Aloja E., Cossu G., Calò S., Palumbo G., Bhugra D. (2014). Eating disorders as a public health issue: prevalence and attributable impairment of quality of life in an Italian community sample.. Int. Rev. Psychiatry.

[31] Carta M.G., Moro M.F., Aguglia E., Balestrieri M., Caraci F., Dell’Osso L., Di Sciascio G., Drago F., Hardoy M.C., D’Aloja E., Machado S., Roncone R., Faravelli C. (2015). The attributable burden of panic disorder in the impairment of quality of life in a national survey in Italy.. Int. J. Soc. Psychiatry.

[32] Carta M.G., Sorbello O., Moro M.F., Bhat K.M., Demelia E., Serra A., Mura G., Sancassiani F., Piga M., Demelia L. (2012). Bipolar disorders and Wilson’s disease.. BMC Psychiatry.

[33] Carta M.G., Moro M.F., Lorefice L., Picardi A., Trincas G., Fenu G., Cocco E., Floris F., Bessonov D., Akiskal H.S., Marrosu M.G. (2014). Multiple sclerosis and bipolar disorders: the burden of comorbidity and its consequences on quality of life.. J. Affect. Disord..

[34] Carta M.G., Lecca M.E., Saba L., Sanfilippo R., Pintus E., Cadoni M., Sancassiani F., Moro M.F., Craboledda D., Lo Giudice C., Finco G., Musu M., Montisci R. (2015). Patients with carotid atherosclerosis who underwent or did not undergo carotid endarterectomy: outcome on mood, cognition and quality of life.. BMC Psychiatry.

[35] Carta M.G., Conti A., Lecca F., Sancassiani F., Cossu G., Carruxi R., Boccone A., Cadoni M., Pisanu A., Francesca Moro M., Demelia L. (2015). The Burden of Depressive and Bipolar Disorders in Celiac Disease.. Clin. Pract. Epidemiol. Ment. Health.

[36] Kessler R.C., Abelson J., Demler O., Escobar J.I., Gibbon M., Guyer M.E., Howes M.J., Jin R., Vega W.A., Walters E.E., Wang P., Zaslavsky A., Zheng H. (2004). Clinical calibration of DSM-IV diagnoses in the World Mental Health (WMH) version of the World Health Organization (WHO) Composite International Diagnostic Interview (WMHCIDI).. Int. J. Methods Psychiatr. Res..

[37] World Health Organization (1997). Composite International Diagnostic Interview (CIDI, Version 2.1)..

[38] Fineberg N.A., Hengartner M.P., Bergbaum C., Gale T., Rössler W., Angst J. (2013). Lifetime comorbidity of obsessive-compulsive disorder and sub-threshold obsessive-compulsive symptomatology in the community: impact, prevalence, socio-demographic and clinical characteristics.. Int. J. Psychiatry Clin. Pract..

[39] McCraw S., Parker G. (2017). The prevalence and outcomes of exposure to potentially traumatic stressful life events compared across patients with bipolar disorder and unipolar depression.. Psychiatry Res..

[40] Sommer J.L., Mota N., Edmondson D., El-Gabalawy R. (2018). Comorbidity in illness-induced posttraumatic stress disorder *versus* posttraumatic stress disorder due to external events in a nationally representative study.. Gen. Hosp. Psychiatry.

